# Supplementary material for: Structure of the T=13 capsid of infectious pancreatic necrosis virus (IPNV)—a salmonid birnavirus
Source: J Virol. 2025 Jan 16;99(2):e01454-24. doi: 10.1128/jvi.01454-24 (PMC11853034; doi:10.1128/jvi.01454-24)
Supplement: Supplemental material — Figures S1 to S6 and S8; Tables S1 to S4. [file jvi.01454-24-s0002.pdf]

Supplemental materials

Supplementary Fig. S1 Cryo-EM sample and cryo-EM single particle analysis of the IPNV L5 particles. A) SDS-PAGE gel image of the purified IPNV L5 particles. Fractions 5-9 (yellow highlighted box, 25 - 35 % (w/v) sucrose concentration) showed all structural proteins (VP1, VP2/pVP2 and VP3) and were pooled for further cryo-EM sample preparation. B) Raw cryo-EM image of the purified IPNV particles. C) FSC curve of the final cryo-EM model of the IPNV. The estimated resolution of the model was calculated to be 2.75 Å by a FSC 0.143 cutoff. D) Calculated local resolution of X, Y, and Z planes. The resolution was scaled by colors in the color panel.

Supplementary Fig. S2 Backbone and side chain fitting of the IPNV atomic model to the cryo-EM reconstructed model (empty/filled). The backbone fitting is shown at contour levels of 1σ and 2σ of the cryo-EM model. Side chain fitting is shown in the region of the surface loops (P domain), C-terminal extensions, and the 5-fold pore.

Supplementary Fig. S3 Cryo-EM model of the filled T=13 IPNV. The atomic model of 13 VP2s fitted to the IPNV empty/filled model (PDB ID: 9GG2) is used to validate structural differences between the empty/filled and filled models. A) Cryo-EM model generated using only filled particles. The 13 VP2 subunits in the icosahedral asymmetric unit are individually colored. B) Gold standard FSC of the

filled IPNV cryo-EM model, with a resolution calculated at 2.78 Å (FSC cutoff 0.143). C) Backbone fitting of the 13 VP2s in the icosahedral asymmetric unit. D) Backbone fitting displayed at contour levels of 1σ and 2σ of the filled IPNV cryo-EM model. E) Side chain fitting shown in the region of the 5-fold pore. F) Complex structure of the 5-fold pore generated from the filled model and the filled/empty model. The superimposition of the two model is also shown.

Supplementary Fig. S4 The close-up views of the N- and C-terminal regions in subunits (a), (b) and (c). The N-terminal and C-terminal regions are colored orange and light blue, respectively.

Supplementary Fig. S5 Amino acid sequence alignment of IBDV-Gx, IBDV-Gt and IPNV-L5 VP2s. The variable loop (red), surface loop 1, 2, 3, 4, and 4' (light blue) and C-terminal extension (orange) are highlighted in the alignment. The alignment was generated using Jalview program (80).

Supplementary Fig. S6 Intrasubunit hydrogen bonds and hydrophobic interfaces in the C-terminal regions of subunits (a), (b) and (c). The BaveSAS and BaveSES values of the hydrophobic interfaces in the C-terminal regions are calculated and presented. Amino acid residues likely contributing significantly to the hydrophobic interactions with the C-terminal extension are shown in spheres in subunit (a)' and (b). A) C-terminal interlocking between subunit (a) (orange) and subunit (a)' (orange red). An intrasubunit hydrogen bond is formed between K12

and N428 residues. B) C-terminal interlocking between subunits (b) (light blue) and (c) (pink). Intrasubunit hydrogen bonds are formed between K12 and N428/E429 residues in subunit (c) and between R425 and N428 residues in subunit (c).

Supplementary Fig. S7 Amino acid sequence alignment of IPNV VP2s using database-registered IPNV strains and IPNV-L5 (Query_11627606). The alignment was generated via BLAST protein search (https://blast.ncbi.nlm.nih.gov/Blast.cgi). Only C-terminal regions (residues 420-425) and the amino acid variations are shown.

Supplementary Fig. S8 shows a structural comparison of the assembled five subunit (a)s at each 5-fold axis between the T=1 subviral particle and the T=13 infectious particle of the IPNV. To compare the pore size, residues 110-117 (variable loops) were removed. The exterior and interior views of the 5-fold region are shown. The distance of two N428 residues (Cα atoms) is used to calculate the radius (R) of the pore. The C-terminal extension region of the T=13 IPNV capsid is colored in orange.

Supplementary Table S1 Dali search result of the jelly-roll structure of the IPNV. The similar jellyroll fold structure of the IPNV VP2 S domain (residues 32-176 and 343-386) was searched in the Dali protein structure comparison server. Capsid protein structural homologues that show a structural similarity with a high

Z-score (> 10) are listed. The same capsid protein homologues were hitted when the entire VP2 structure of the IPNV was searched.

Supplementary Table S2 Capsid structures of IPNV and IBDV birnaviruses obtained from cell culture or recombinantly expressed VP2/preVP2 proteins. The table is generated based on results from this study as well as previously reported studies (20-22,24,29).

Supplementary Table S3 Twelve amino acid alternations of IPNV VP2 from the virulent AY379740 strain.

Supplementary Table S4 Validation statistics of cryo-EM reconstruction and atomic modeling.

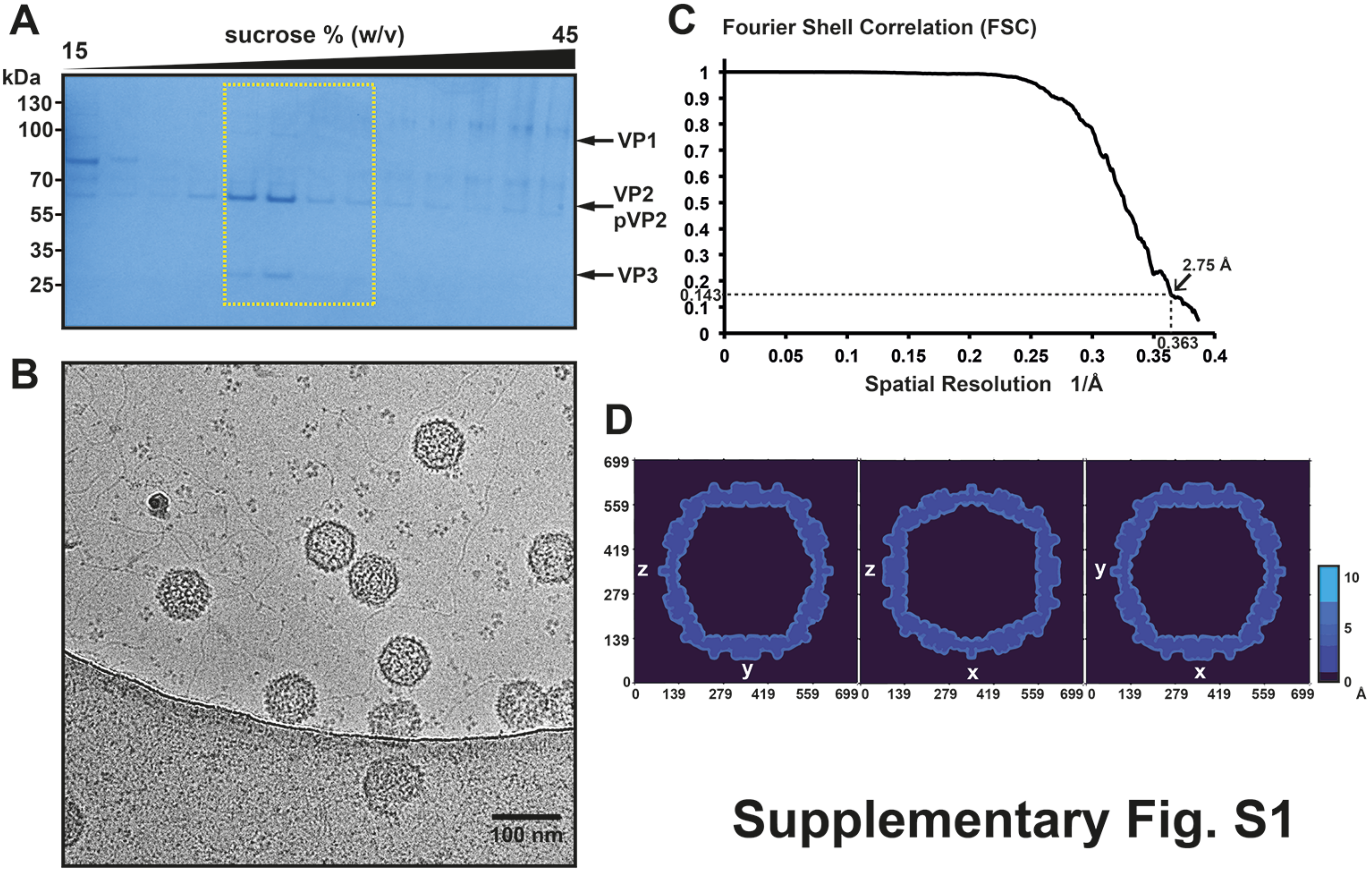

Supplementary Fig. S1

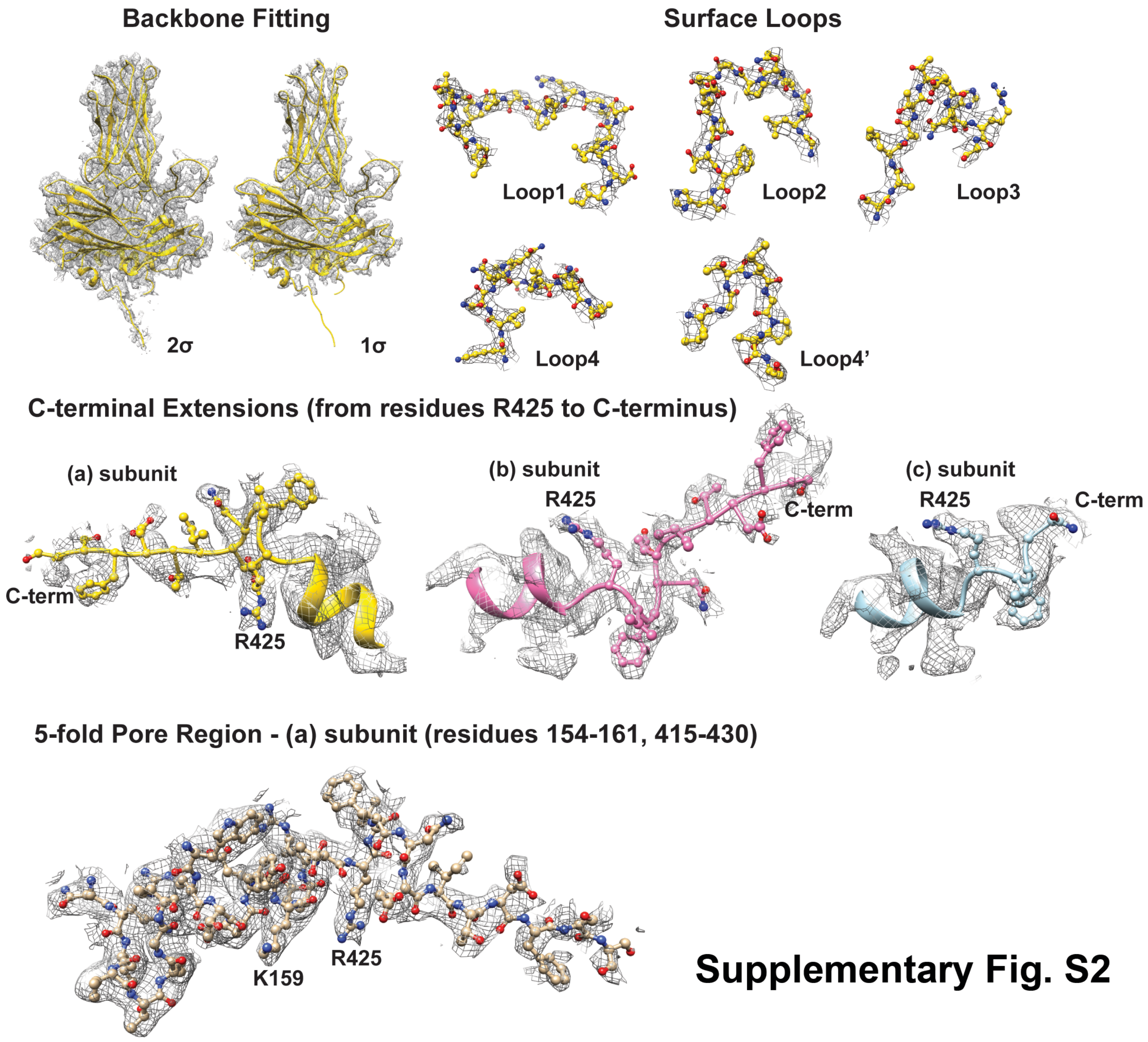

Supplementary Fig. S2

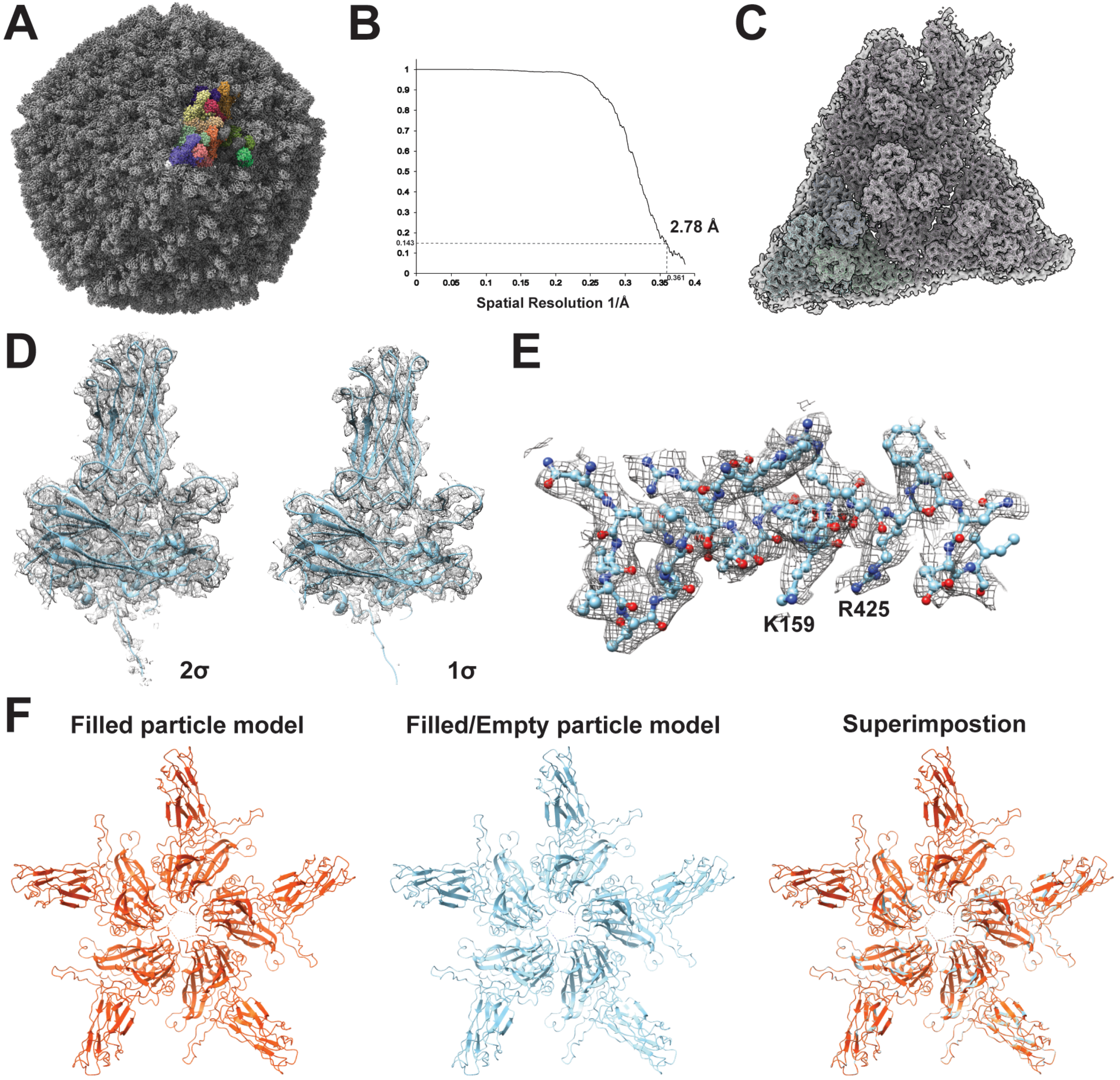

Supplementary Fig. S3

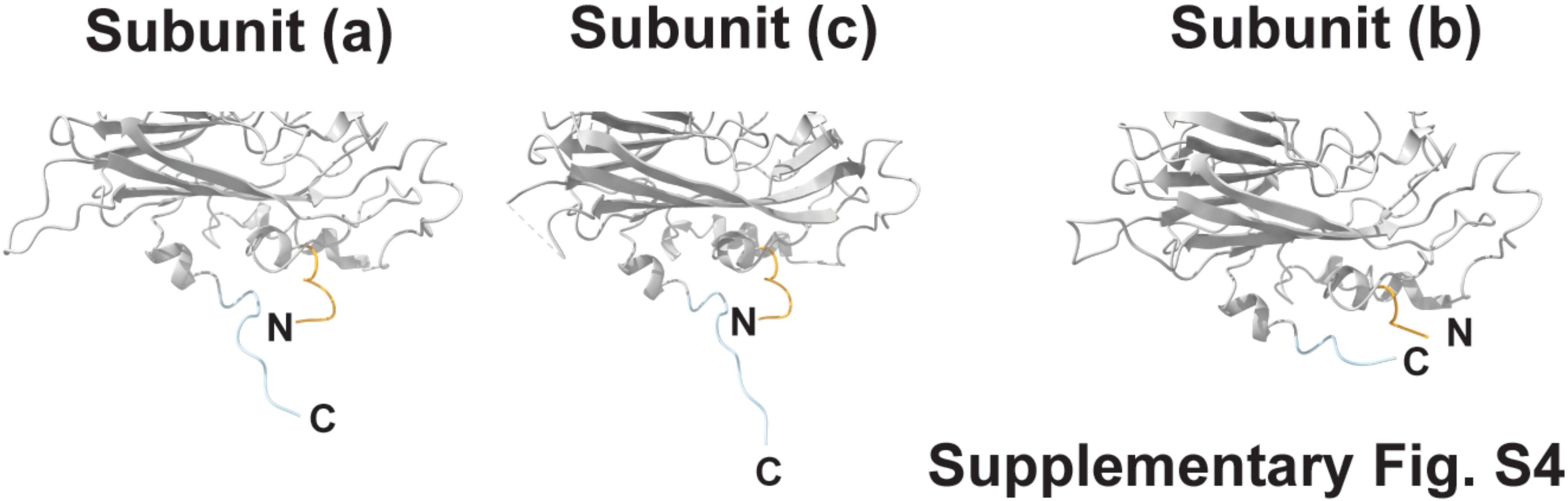

Supplementary Fig. S4

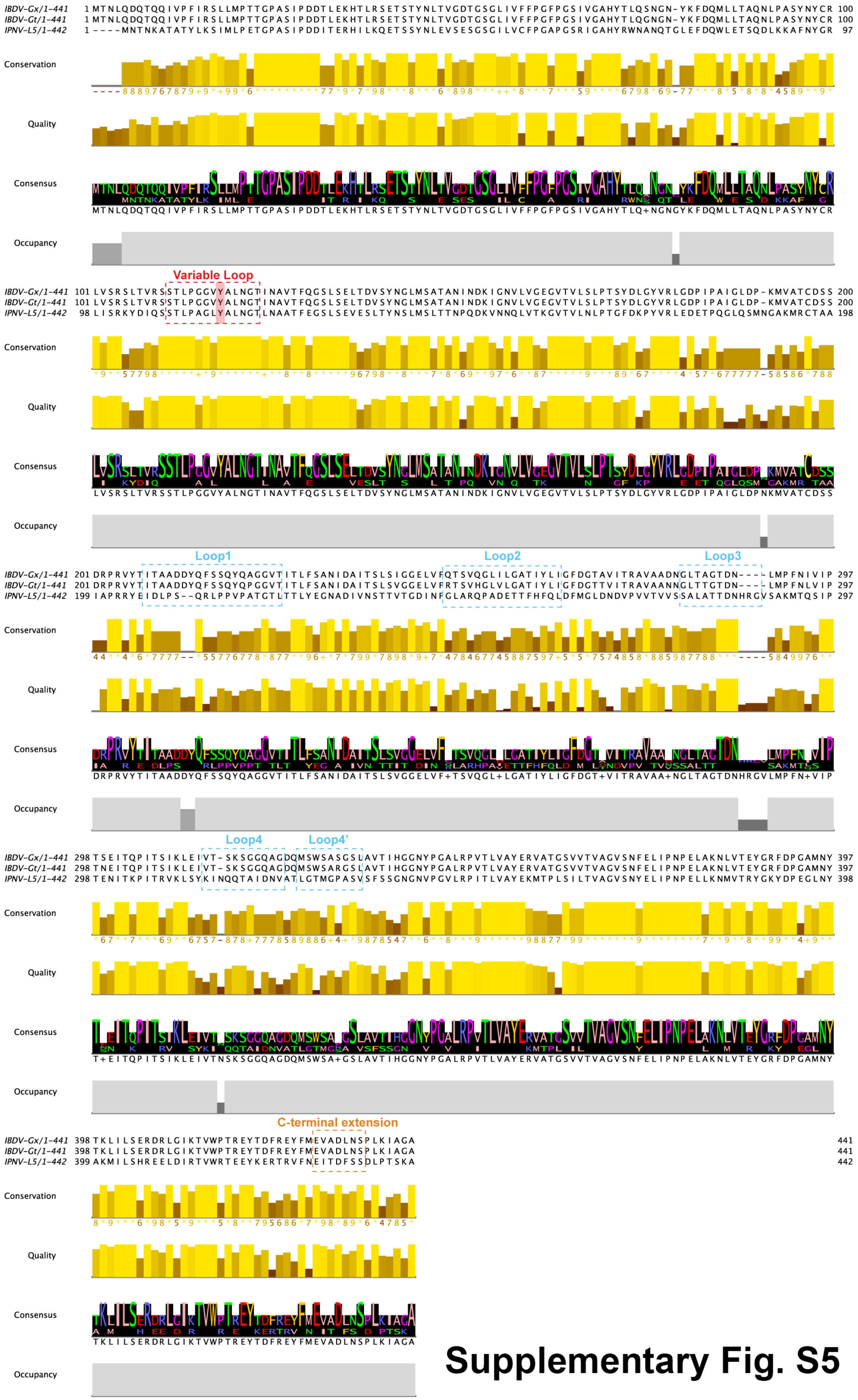

Supplementary Fig. S5

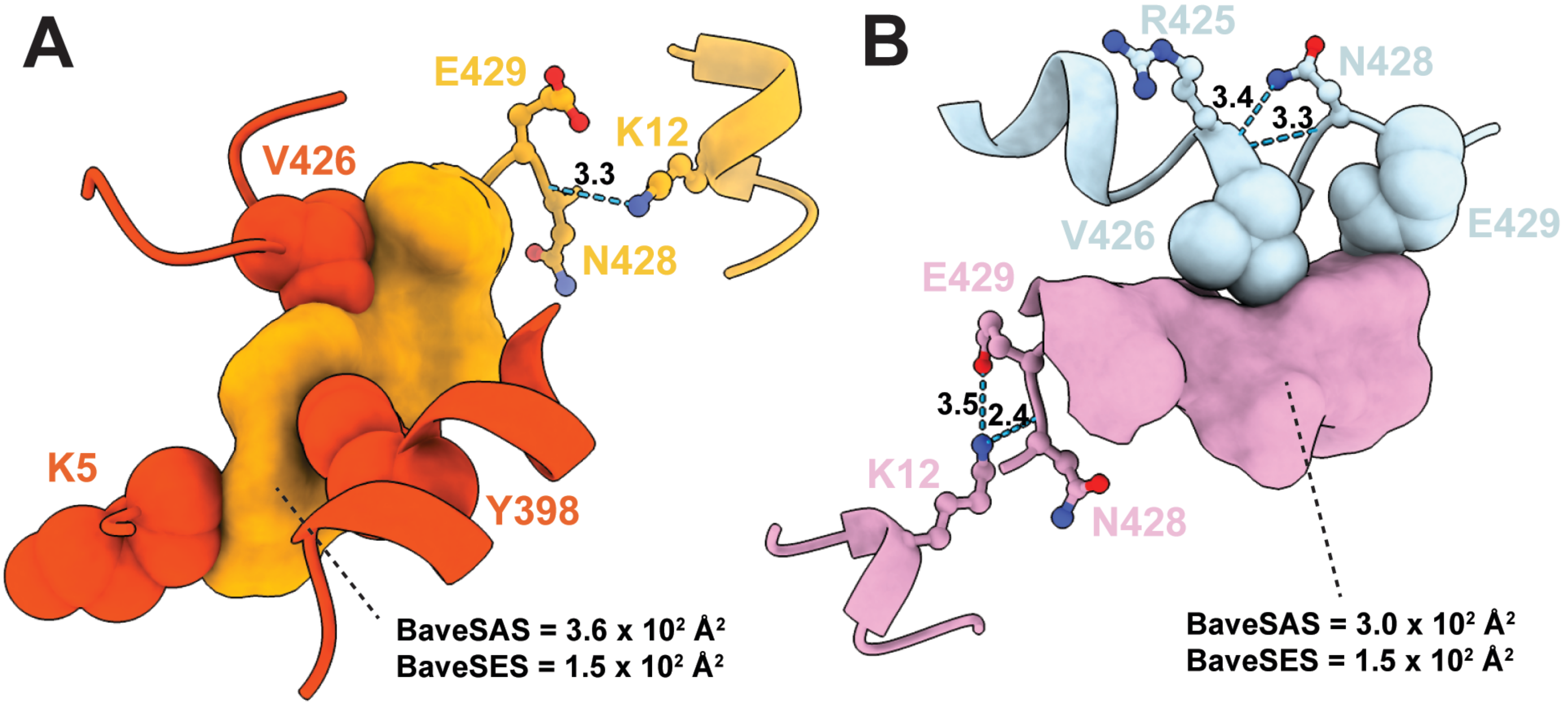

Supplementary Fig. S6

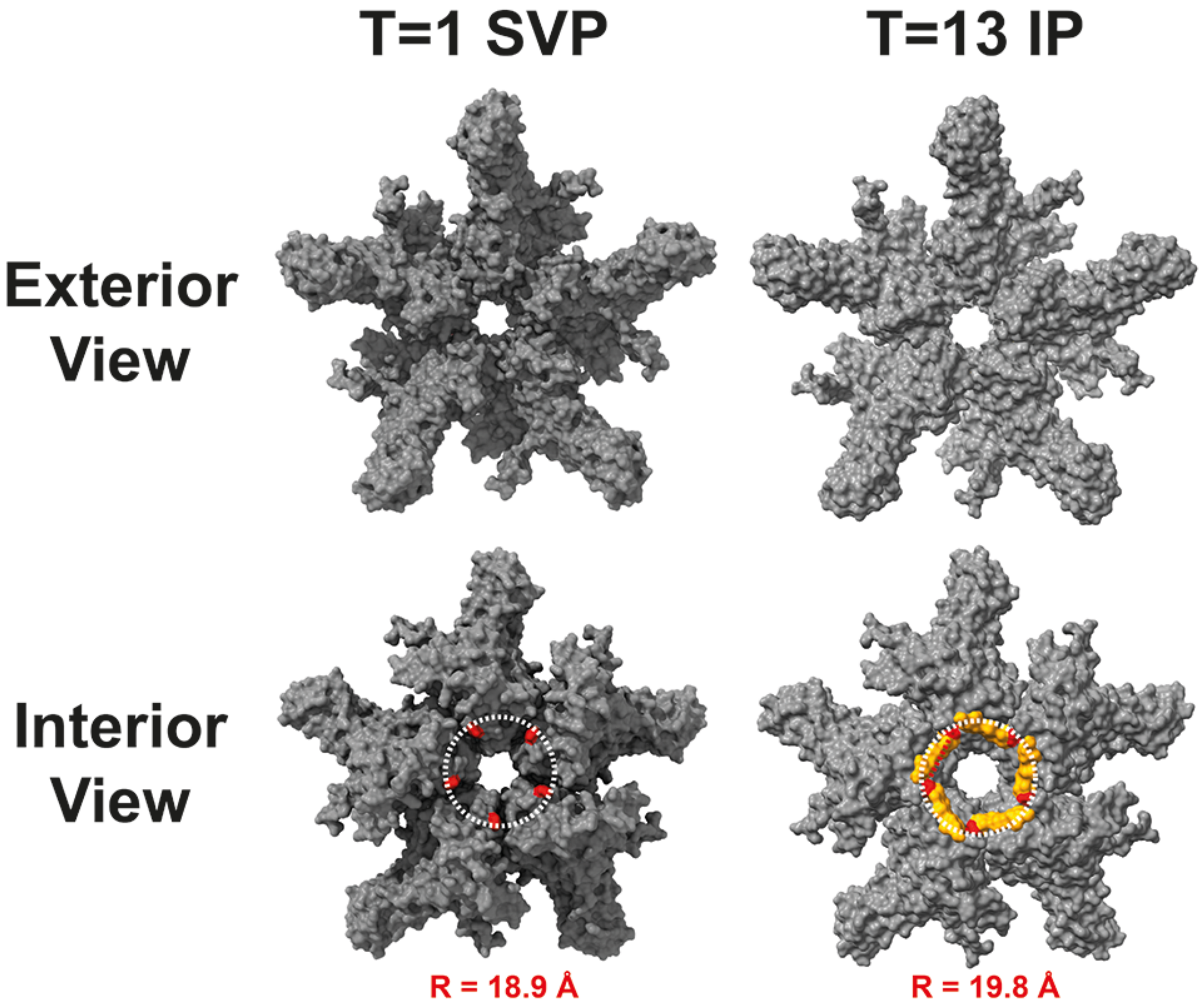

Supplementary Fig. S8

Supplemenatry Table S1

Virus Strain	Order	Family	PDB ID	Z-score
Infectious bursal disease virus	none	Birnaviridae	2QSY	24.6
Lake Sinai virus	Nodamuvirales	Sinhaliviridae	7XPG	12.6
Nudaurelia capensis omega virus	none	Tetraviridae	8AAY	11.9
Pariacoto virus	Nodamuvirales	Nodaviridae	1F8V	11.1

Supplementary Table S2

Cell Culture	Capsid Structure	Reference
IPNV virion	T =13	This sudy
IBDV virion	T =13	Bao et al., 2022
**Recombinant Protein**	**Capsid Structure**	**Reference**
IPNV VP2 (1-442)	T=1	Coulibaly et al 2010
IBDV VP2 (1-442)	T=1	Coulibaly et al 2005
IBDV preVP2 (1-452)	T=1	Garriga et al 2006
IBDV preVP2 (1-466)	T=1 and T=13	Saugar et al 2010
IBDV preVP2 (11-466)	Tubular or irregular shape	Saugar et al 2010

Supplementary Fig. S3

Amino acid position	AY379740	L5
217	T	P
221	A	T
245	S	G
247	T	A
248	E	R
252	V	D
255	K	T
257	D	H
278	V	A
282	N	T
285	Y	H
321	G	D

Supplementary Table S4

	IPNV-L5
**Data collection**	
Maganification	81 000
Voltage (kV)	300
Electron exposure (e-/Å^2)	26.8
Defocus range (μm)	-1.7 to -0.5
Pixel size (Å/pixel)	1.058 (1.284)
Symmetry	I
Final particle (No.)	35 498
Map resolution (Å)	2.75
FSC threshold	0.143
**Model composition and quality**	
Non-hydrogen atoms	42 492
Protein residues	5 500
B-factors (Å^2)	
Protein (min/max/mean)	36.7/135.7/55.85
RMSD	
Bond length (Å)	0.007
Bond Angles (°)	0.864
Validation	
MolProbity score	2.19
Clashscore	12.04
Poor rotamer (%)	0.17
Ramachandran plot	
Favored (%)	88.34
Allowed (%)	11.37
Outliers (%)	0.29
CC (mask)	0.87
EMRinger score	3.23
Data depostion	
EMDB	51321
PDB	9GG2
